# Supplementary material for: Transfer functions linking neural calcium to single voxel functional ultrasound signal
Source: Nat Commun. 2020 Jun 11;11:2954. doi: 10.1038/s41467-020-16774-9 (PMC7290037; doi:10.1038/s41467-020-16774-9)
Supplement: Supplementary file 1 — Supplementary Information [file 41467_2020_16774_MOESM1_ESM.pdf]

Supplementary Informations

Transfer functions linking neural calcium to single voxel functional ultrasound signal,

Aydin et al., Nat Commun, 2020

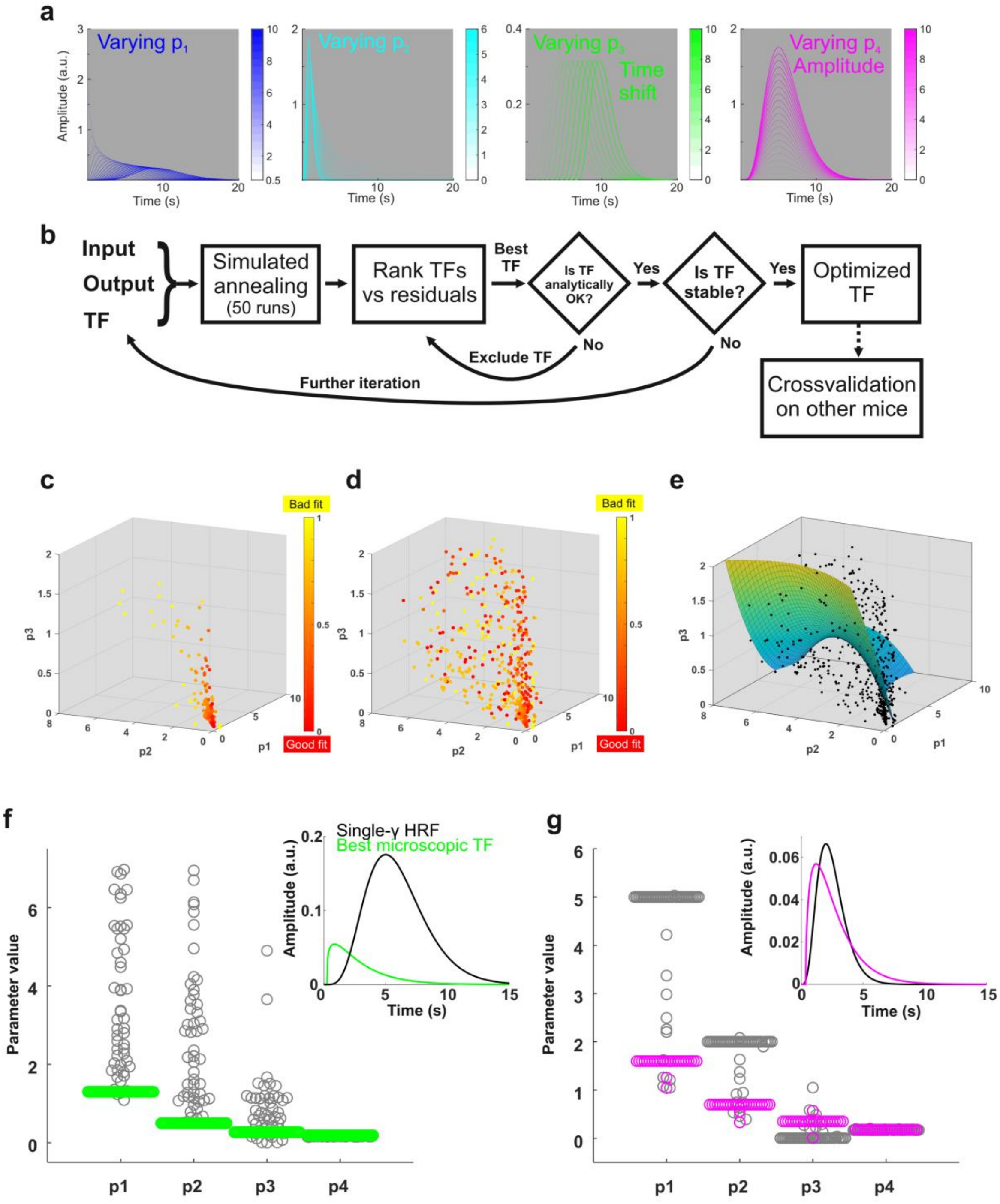

Supplementary Fig.1 Legend on the next page

Supplementary Fig. 1 Optimization of the microscopic transfer function (μTF)

(**a**) The initial single gamma TF was taken from SPM12, removing the second component (undershoot) and adding a time-shift parameter, thus making a total of 4 free parameters. Each parameter modifies the TF shape as shown in the panels. The color scale (Z axis) is the increasing value of the parameter. Y axis is the amplitude of the TFs generated while increasing the value of the parameter. Note that p_4 (scaling factor) has a major effect on amplitude, p_3 on the onset, p_1 and p_2 affecting the whole dynamics of the TF. (**b**) The algorithm used in the optimization process: with a machine learning optimization (simulated annealing), the same set of initial values (cost function fixed) result in many optimized TFs, then ranked with respect to their prediction quality. The best TF of the iteration is checked to be analytically acceptable (test on shape, see Methods) or discarded and the next TF in the rank is analyzed and so on. When a TF passes this first check, it is then tested for its stability over a high number of optimization runs (> 40). If the check on the shape eliminates too many TFs or if the TF is not stable over most of the optimization runs, its p_1-p_4 parameters are passed to another iteration process. (**c**) The optimized TFs found with simulated annealing can be represented in terms of their first 3 parameters, (p_4 is a scaling factor) and the quality of the fit can be expressed as the inverse of the normalized residuals, color coded. The plot shows 200 optimization runs for a single mouse. Good fit parameters tend to accumulate close to the origin of the p_1-p_2 axes. (**d**) Merging the same data with those from 5 mice. (**e**) 3D fit of order 2 approximates well the residual distribution (fit weighted by the inverse of the residuals). It makes further optimization faster to calculate by restricting the 3D space to a portion mapped by the hyper-plane. (**f**) Iteration optimization process for the microscopic TF: dispersion of the final value parameters set after optimization starting from the initial parameters of the single gamma-component HRF (gray curve in inset) is represented by grey circles (same p_1-p_3 values as in (**c**)). Each optimization run produces a final set, corresponding to a local minimum, usually different from the initial one. In case the local minimum is very stable, the initial and final sets are identical. A second optimization iteration consists in 50 other runs, using the best fit from the previous iteration as the starting value set (green circles). The best fit (green curve in inset) is the one with the lowest residual and analytically acceptable (see Methods). All the green circles are stable, i.e., they do not vary after a new optimization process. Parameter stability over many optimization runs is a mandatory requirement to define a TF as optimal for a given dataset. In the inset, the gray curve represents the single gamma HRF while the green one is the best microscopic TF. (**g**) The choice of the initial value for the first iteration is important: starting from a function with parameters distant from those of the best TF, as in panel (**f**), results in heterogeneous fits, some bad and some possibly good. Choosing, instead, a starting TF ‘close’ to the best one (gray curve in inset) does not provide much heterogeneity in the fits and limits the improvement obtained with a second iteration (pink curve in the inset, similar to the best microscopic TF, but with a worse prediction performance). Source data are provided as a Source Data file.

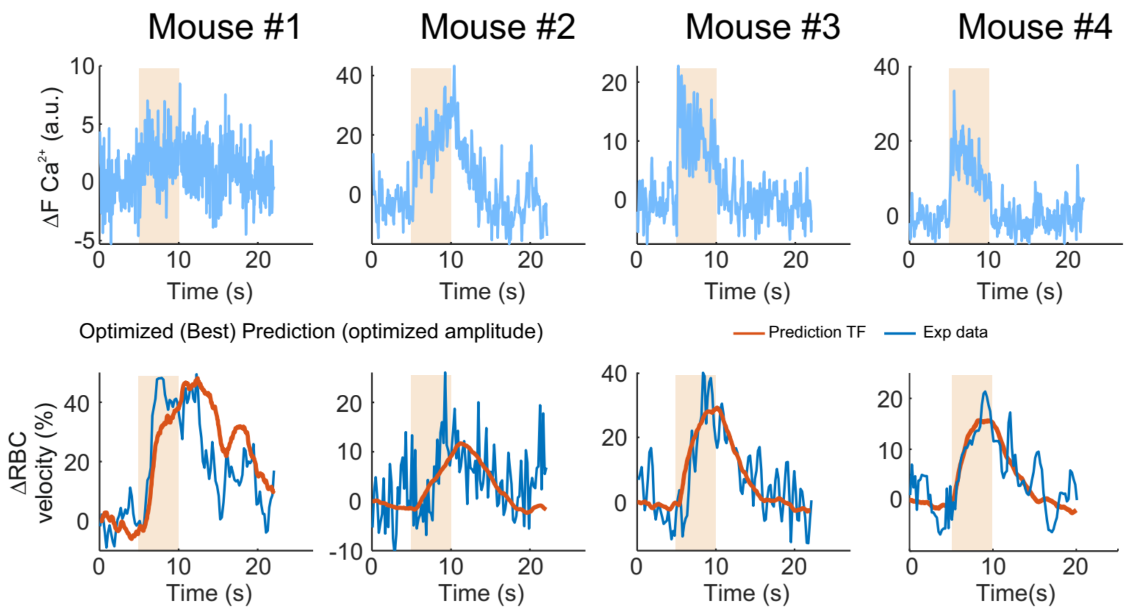

Supplementary Fig. 2 Vascular response predictions to isoamyl acetate (AA), using the ET-based μTF

Ca^{2+} and RBC velocity responses to AA (1 %, 5 s). Although the μTF was optimized on responses to ET, it provided good predictions across animals (Pearson coefficient in 4 mice: 0.77, 0.59, 0.86, 0.82).

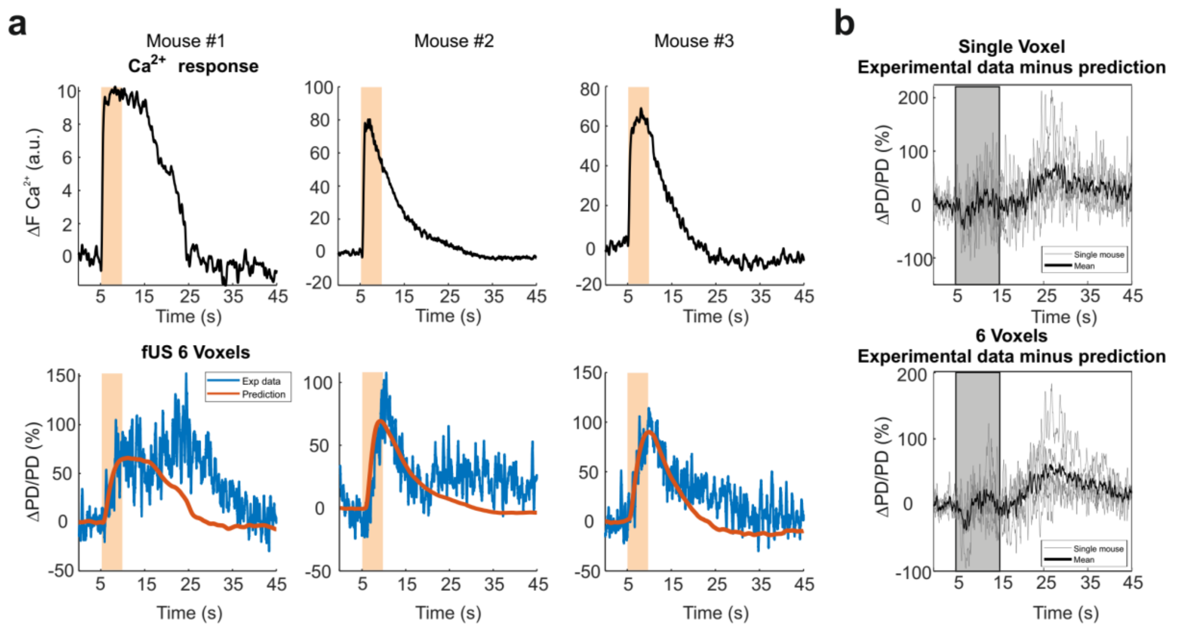

Supplementary Fig. 3 At high odor concentration, the mTF reveals a delayed secondary component at a mesoscopic scale

(**a**) Ca^{2+} (top) and fUS (bottom) responses to 6 % ET (5 s) for 3 mice. Vascular responses are not correctly (orange traces) predicted with the mTF. (**b**) fUS vascular responses (single and 6 voxels) after subtraction of their prediction using the mTF. As at the microscopic level, traces fluctuations between 5 and 15 s (gray background) result from slight difference of onset, slope, and response peak between real and predicted responses. The fUS secondary component is delayed by ~10-15 s.

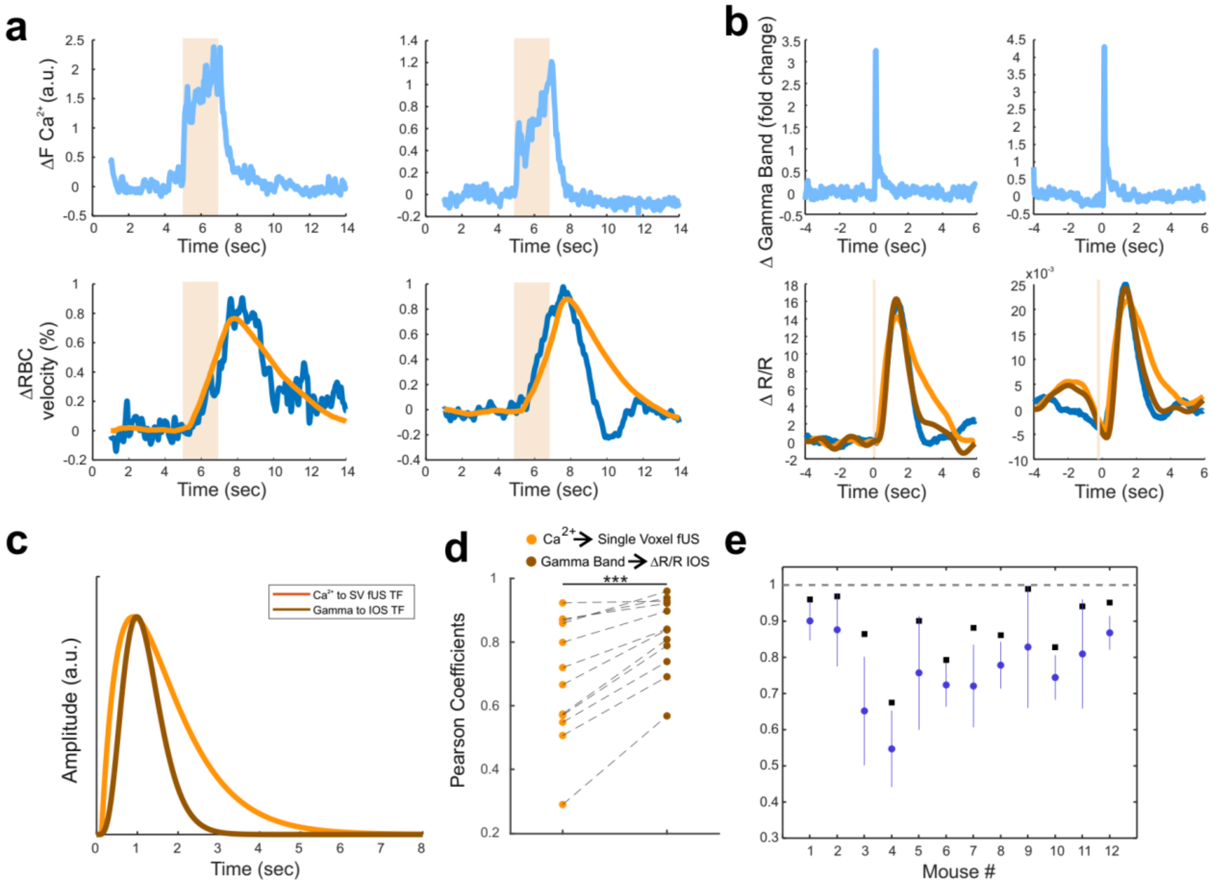

Supplementary Fig. 4 Application of the TFs to the OB and the somatosensory cortex of awake mice

(**a**) Ca^{2+} (top) and RBC velocity (bottom) responses to mild odor concentration (orange background) in the OB of awake mice. Data from Rungta et al., 2018 [1]. (**b**) Gamma-band LFP (top) and IOS reflection changes (bottom) from the somatosensory of awake mice. Data from Winder et al., 2017 [2]. Orange traces are the prediction made by our mTF, brown traces are the predictions made by a new TF optimized on these data (see below). (**c**) Comparison between the two TFs. (**d**) Prediction quality of both TFs (n=12 mice). Note the improvement with the new TF (paired two-sided t-test, $p=8.10^{-5}$). (**e**) As in Fig.1d, quantification of the prediction robustness (mean ± SD, n=12 mice) for each TF, either on the data used to compute it (same mouse, self-validation, black square) or on other mice's data (cross-validation, blue symbols). Source data are provided as a Source Data file.

References

1. Rungta,R.L., Chaigneau,E., Osmanski,B.F., & Charpak,S. Vascular Compartmentalization of Functional Hyperemia from the Synapse to the Pia. *Neuron* **99**, 362-375 (2018).
2. Winder,A.T., Echagarruga,C., Zhang,Q., & Drew,P.J. Weak correlations between hemodynamic signals and ongoing neural activity during the resting state. *Nat. Neurosci* **20**, 1761-1769 (2017).
