# Supplementary material for: Transfer functions linking neural calcium to single voxel functional ultrasound signal
Source: Nat Commun. 2020 Jun 11;11:2954. doi: 10.1038/s41467-020-16774-9 (PMC7290037; doi:10.1038/s41467-020-16774-9)
Supplement: Supplementary file 3 — Reporting Summary [file 41467_2020_16774_MOESM3_ESM.pdf]

Corresponding author(s): Serge Charpak & Davide Boido

Last updated by author(s): 03/05/2020

Reporting Summary

Nature Research wishes to improve the reproducibility of the work that we publish. This form provides structure for consistency and transparency in reporting. For further information on Nature Research policies, see Authors & Referees and the Editorial Policy Checklist.

Statistics

For all statistical analyses, confirm that the following items are present in the figure legend, table legend, main text, or Methods section.

n/a	Confirmed	
☐	☒	The exact sample size (*n*) for each experimental group/condition, given as a discrete number and unit of measurement
☐	☒	A statement on whether measurements were taken from distinct samples or whether the same sample was measured repeatedly
☐	☒	The statistical test(s) used AND whether they are one- or two-sided<br>*Only common tests should be described solely by name; describe more complex techniques in the Methods section.*
☒	☐	A description of all covariates tested
☐	☒	A description of any assumptions or corrections, such as tests of normality and adjustment for multiple comparisons
☐	☒	A full description of the statistical parameters including central tendency (e.g. means) or other basic estimates (e.g. regression coefficient) AND variation (e.g. standard deviation) or associated estimates of uncertainty (e.g. confidence intervals)
☐	☒	For null hypothesis testing, the test statistic (e.g. *F*, *t*, *r*) with confidence intervals, effect sizes, degrees of freedom and *P* value noted<br>*Give P values as exact values whenever suitable.*
☒	☐	For Bayesian analysis, information on the choice of priors and Markov chain Monte Carlo settings
☒	☐	For hierarchical and complex designs, identification of the appropriate level for tests and full reporting of outcomes
☒	☐	Estimates of effect sizes (e.g. Cohen's *d*, Pearson's *r*), indicating how they were calculated

Our web collection on statistics for biologists contains articles on many of the points above.

Software and code

Policy information about availability of computer code

Data collection	Custom made LabVIEW 2013 software was used to collect 2P data. Custom made Matlab 2012 software was used to collect fUS data. Custom made LabVIEW 2009 software was used to drive the olfactometer.
Data analysis	2P data were analyzed using custom made LabView 2013 and Matlab 2018a softwares and scripts. fUS data were analyzed using Malatb 2018a softwares. TF were computed using Matlab 2018a custom scripts. The whole code used to analyze the datasets and get the results and figures is available from the corresponding authors on reasonable request. As described in the Methods section, a simple version of our scripts, with a graphical user interface, and a subset of our data is available on GitLab (https://gitlab.com/AliK_A/buildtf) and on Zenodo (https://doi.org/10.5281/zenodo.3773863).

For manuscripts utilizing custom algorithms or software that are central to the research but not yet described in published literature, software must be made available to editors/reviewers. We strongly encourage code deposition in a community repository (e.g. GitHub). See the Nature Research guidelines for submitting code & software for further information.

Data

Policy information about availability of data

All manuscripts must include a data availability statement. This statement should provide the following information, where applicable:

- Accession codes, unique identifiers, or web links for publicly available datasets
- A list of figures that have associated raw data
- A description of any restrictions on data availability

The datasets generated and analyzed during the current study are available from the corresponding authors on reasonable request. A subset of our data is available on Zenodo (https://doi.org/10.5281/zenodo.3773863) to test our software. The source data underlying Figs 1d, 4c, 5a-b, Table 1 and Supplementary Figs 1c-d, 1f-g, 4d-e are provided as a Source Data file.

Field-specific reporting

Please select the one below that is the best fit for your research. If you are not sure, read the appropriate sections before making your selection.

☒ Life sciences ☐ Behavioural & social sciences ☐ Ecological, evolutionary & environmental sciences

For a reference copy of the document with all sections, see nature.com/documents/nr-reporting-summary-flat.pdf

Life sciences study design

All studies must disclose on these points even when the disclosure is negative.

Sample size	Sample sizes were chosen to be consistent with previous studies (Boido et al., Nat Commun, 2019).
Data exclusions	Some data were excluded following pre-established exclusion criteria: when sedation decreased, i.e. upon animal movement, whisking or irregular respiration frequency. These data were readily discarded.
Replication	Reliability over time (days-weeks and during the experiment) was assessed for each techniques in a previous study under the same conditions (Boido et al., Nat Commun, 2019). For the current work, the Transfer Function reproducibility was assessed, by means of the Pearson Coefficient throughout the whole study for different stimulus modulation (see Fig.2) and across mice (see Fig.1).
Randomization	All mice underwent the same protocol, so randomization was not necessary. Odor stimulations were delivered in an interleaved manner inside each experiment.
Blinding	There was no blindind to group allocation since there was not any group allocation in this study. Analyses have been done with automated scripts with minimal user input.

Reporting for specific materials, systems and methods

We require information from authors about some types of materials, experimental systems and methods used in many studies. Here, indicate whether each material, system or method listed is relevant to your study. If you are not sure if a list item applies to your research, read the appropriate section before selecting a response.

Materials & experimental systems

n/a	Involved in the study	
☒	☐	Antibodies
☒	☐	Eukaryotic cell lines
☒	☐	Palaeontology
☐	☒	Animals and other organisms
☒	☐	Human research participants
☒	☐	Clinical data

Methods

n/a	Involved in the study	
☒	☐	ChIP-seq
☒	☐	Flow cytometry
☒	☐	MRI-based neuroimaging

Animals and other organisms

Policy information about studies involving animals; ARRIVE guidelines recommended for reporting animal research

Laboratory animals	Thy1-GCaMP6f (GP5.11) [C57BL/6J-Tg(Thy1-GCaMP6f)GP5.11Dkim/J] crossed with M72->S50-IRES-hChRVenus, both male and female, from 3 to 12 months old, at 24 °C and 50% humidity.
Wild animals	No wild animals were used in this study.
Field-collected samples	No field collected samples were used in this study.
Ethics oversight	All animal care and experiments were performed in accordance with the INSERM Animal Care and Use Commitee Guidelines (protocol number CEEA34.SC.122.12 and CEEA34.SC.123.12).

Note that full information on the approval of the study protocol must also be provided in the manuscript.
